# Supplementary material for: The Portal Venous Pulsatility Index and Main Portal Vein Diameter as Surrogate Markers for Liver Fibrosis in Nonalcoholic Fatty Liver Disease and Metabolic-Dysfunction-Associated Steatotic Liver Disease
Source: Diagnostics (Basel). 2024 Feb 11;14(4):393. doi: 10.3390/diagnostics14040393 (PMC10888470; doi:10.3390/diagnostics14040393)
Supplement: Supplementary file 1 [file diagnostics-14-00393-s001.zip › diagnostics-2847614-supplementary.pdf]

Supplementary Materials

Table S1. Baseline characteristics of patients with age over 30.

	Total (n = 184)	SF (–) Group (n = 171)	SF (+) Group (n = 13)	p Value
Male sex	178 (96.7)	165 (96.5)	13 (100.0)	1.000
Age	40.0 (35.0, 44.0)	40.0 (35.0, 44.0)	39.0 (34.0, 42.0)	0.260
Height (cm)	174.2 ± 5.7	174.2 ± 5.7	174.0 ± 6.4	0.907
Weight (kg)	83.4 ± 13.0	82.2 ± 12.2	99.0 ± 13.0	<0.001
BMI (kg/m^2	27.4 ± 3.7	27.0 ± 3.3	32.7 ± 4.3	<0.001
DM	26 (14.1)	24 (14.0)	2 (15.4)	1.000
HTN	64 (34.8)	60 (35.1)	4 (30.8)	0.990
Dyslipidemia	115 (62.5)	106 (62.0)	9 (69.2)	0.824
PLT (1000/μL)	260.0 (225.0, 294.2)	260.0 (225.0, 296.0)	257.0 (225.0, 278.0)	0.808
AST (IU/L)	29.0 (22.9, 37.1)	28.8 (22.1, 36.2)	51.0 (27.4, 62.0)	0.003
ALT (IU/L)	42.0 (29.8, 66.3)	41.0 (28.5, 64.0)	84.0 (61.0, 110.0)	<0.001
TB (mg/dL)	0.8 (0.6, 1.0)	0.8 (0.6, 1.1)	0.8 (0.7, 0.9)	0.308
GGT (U/L)	60.0 (35.0, 102.0)	58.0 (34.0, 97.0)	99.0 (65.0, 117.8)	0.053
Albumin (mg/dL)	4.8 (4.7, 5.0)	4.8 (4.7, 5.0)	4.7 (4.6, 4.9)	0.276
PT (INR)	0.98 (0.94, 1.01)	0.98 (0.94, 1.01)	0.96 (0.94, 0.97)	0.081
CAP (dB/m)	290.0 (245.8, 332.0)	281.0 (244.0, 326.5)	356.0 (327.0, 367.0)	<0.001
LSM (kPa)	4.6 (4.0, 5.7)	4.5 (4.0, 5.3)	9.0 (8.7, 11.6)	<0.001

Values are presented as mean ± standard deviation, median (interquartile range), or number (%). NAFLD was stratified by using liver stiffness measurement with the threshold of 8.2kPa. Abbreviations: ALT, alanine aminotransferase; AST, aspartate aminotransferase; BMI, body mass index; CAP, controlled attenuation parameter; DM, diabetes mellitus; GGT, gamma-glutamyl transferase; HTN, hypertension; INR, international normalized ratio; LSM, liver stiffness measurement; NAFLD, nonalcoholic fatty liver disease; PLT, platelet count; PT, prothrombin time; TB, total bilirubin.

Table S2. Factors associated with significant fibrosis in patients with age over 30.

	Crude OR (95% CI)	*p*
VPI (0.01 scale)	0.822 (0.721–0.938)	0.004
MPVD	2.233 (1.391–3.585)	<0.001
BMI (kg/m^2)	1.591 (1.300–1.949)	<0.001
DM	1.114 (0.232–5.338)	0.893
Dyslipidemia	1.380 (0.408–4.662)	0.604
HTN	0.822 (0.243–2.782)	0.753

Abbreviations: BMI, body mass index; DM, diabetes mellitus; HTN, hypertension; MPVD, main portal vein diameter; OR, odds ratio; VPI, portal venous pulsatility index.

Table S3. Sensitivity analysis using different determinant values for significant fibrosis.

		Crude OR (95% CI)	*p*	Age-Adjusted OR (95% CI)	*p*	Multivariate-Adjusted OR (95% CI)	*p*
**FAST score 0.67** SF (−) (*n* = 939) vs. SF (+) (*n* = 55)	VPI (0.01 scale)	0.905 (0.868–0.942)	<0.001	0.885 (0.846–0.925)	<0.001	0.916 (0.874–0.960)	0.002
	MPVD	1.806 (1.501–2.172)	<0.001	1.811 (1.506–2.177)	<0.001	1.578 (1.296–1.920)	<0.001
	BMI ≥ 30 kg/m^2	8.462 (4.589–15.606)	<0.001	8.216 (4.449–15.172)	<0.001		
	Age	0.957 (0.913–1.002)	0.063	-	-	-	-
	DM	1.264 (0.379–4.217)	0.704	-	-	-	-
	Dyslipidemia	2.902 (1.664–5.060)	<0.001	3.657 (2.069–6.463)	<0.001	2.461 (1.355–4.469)	0.003
	HTN	0.904 (0.434–1.884)	0.789	-	-	-	-
**LSM 7.0** SF (−) (*n* = 871) vs. SF (+) (*n* = 123)	VPI (0.01 scale)	0.925 (0.902–0.949)	<0.001	0.914 (0.889–0.939)	<0.001	0.949 (0.922–0.977)	<0.001
	MPVD	1.924 (1.657–2.235)	<0.001	1.931 (1.662–2.243)	<0.001	1.706 (1.451–2.007)	<0.001
	BMI ≥ 30 kg/m^2	10.337 (6.735–15.864)	<0.001	10.250 (6.674–15.743)	<0.001		
	Age	0.983 (0.957–1.009)	0.188	-	-	-	-
	DM	1.360 (0.593–3.122)	0.468	-	-	-	-
	Dyslipidemia	1.387 (0.945–2.036)	0.095	1.547 (1.039–2.306)	0.032	0.884 (0.566–1.381)	0.588
	HTN	1.288 (0.807–2.056)	0.288	-	-	-	-

Multivariate model: BMI ≥ 3 0kg/m^2 and age were adjusted as covariates. Abbreviations: BMI, body mass index; DM, diabetes mellitus; FAST, Fibroscan-AST; HTN, hypertension; LSM, liver stiffness measurement; MPVD, main portal vein diameter; OR, odds ratio; VPI, portal venous pulsatility index.

Table S4. Factors associated with significant fibrosis in MASLD patients (n = 939).

	Crude OR (95% CI)	*p*	Age-Adjusted OR (95% CI)	*p*	Multivariate-Adjusted OR (95% CI)	*p*
VPI (0.01 scale)	0.924 (0.893–0.956)	<0.001	0.914 (0.881-0.948)	<0.001	0.951 (0.924–0.979)	0.001
MPVD	1.743 (1.467–2.071)	<0.001	1.742 (1.467–2.069)	<0.001	1.687 (1.435–1.984)	<0.001
BMI (kg/m^2)	1.381 (1.288–1.479)	<0.001	1.385 (1.291–1.485)	<0.001	-	-
BMI ≥ 30 kg/m^2	14.781 (7.775–28.103)	<0.001	14.853 (7.796–28.298)	<0.001		
Age	0.989 (0.960–1.024)	0.529	-	-	-	-
DM	2.121 (0.863–5.212)	0.101	2.484 (0.964–6.400)	0.060	1.002 (0.397–2.528)	0.996
Dyslipidemia	1.523 (0.928–2.500)	0.096	1.655 (0.990–2.765)	0.055	0.854 (0.548–1.331)	0.487
HTN	0.924 (0.484–1.763)	0.810	-	-	-	-

Multivariate model: BMI≥30kg/m2 and age were adjusted as covariates. Abbreviations: BMI, body mass index; DM, diabetes mellitus; HTN, hypertension; MPVD, main portal vein diameter; OR, odds ratio; VPI, portal venous pulsatility index.

Table S5. Comparison of baseline characteristics between train and test cohorts.

	Training Cohort (n = 662)	Test Cohort (n = 332)	*p* Value
Male sex	652 (98.5)	322 (97.0)	0.177
Age	21.0 (20.0, 26.0)	21.0 (20.0, 24.3)	0.119
Height (cm)	174.6 ± 5.8	174.6 ± 6.0	0.980
Weight (kg)	83.9 ± 14.8	83.5 ± 15.4	0.676
BMI (kg/m^2)	27.5 ± 4.3	27.3 ± 4.4	0.625
DM	33 (5.0)	11 (3.3)	0.296
HTN	131 (19.8)	45 (13.6)	0.019
Dyslipidemia	239 (36.1)	114 (34.3)	0.632
PLT (1000/μL)	267.0 (234.0, 305.0)	264.5 (232.0, 292.0)	0.521
AST (IU/L)	28.5 (20.4, 40.0)	28.8 (21.2, 43.6)	0.965
ALT (IU/L)	45.5 (23.0, 81.0)	50.0 (25.0, 87.0)	0.936
TB (mg/dL)	0.8 (0.6, 1.0)	0.8 (0.7, 1.0)	0.718
GGT (U/L)	40.0 (23.0, 70.5)	40.0 (22.0, 68.0)	0.879
Albumin (mg/dL)	4.9 (4.7, 5.1)	4.9 (4.7, 5.1)	0.879
PT (INR)	1.01 (0.97, 1.05)	1.01 (0.97, 1.05)	0.950
CAP (dB/m)	276.0 (242.2, 329.0)	280.0 (241.5, 332.0)	0.588
LSM (kPa)	4.8 (4.0, 6.0)	4.8 (4.0, 5.8)	0.541

Values are presented as mean ± standard deviation, median (interquartile range), or number (%). Abbreviations: ALT, alanine aminotransferase; AST, aspartate aminotransferase; BMI, body mass index; CAP, controlled attenuation parameter; DM, diabetes mellitus; GGT, gamma-glutamyl transferase; HTN, hypertension; INR, international normalized ratio; LSM, liver stiffness measurement; NAFLD, nonalcoholic fatty liver disease; PLT, platelet count; PT, prothrombin time; TB, total bilirubin.

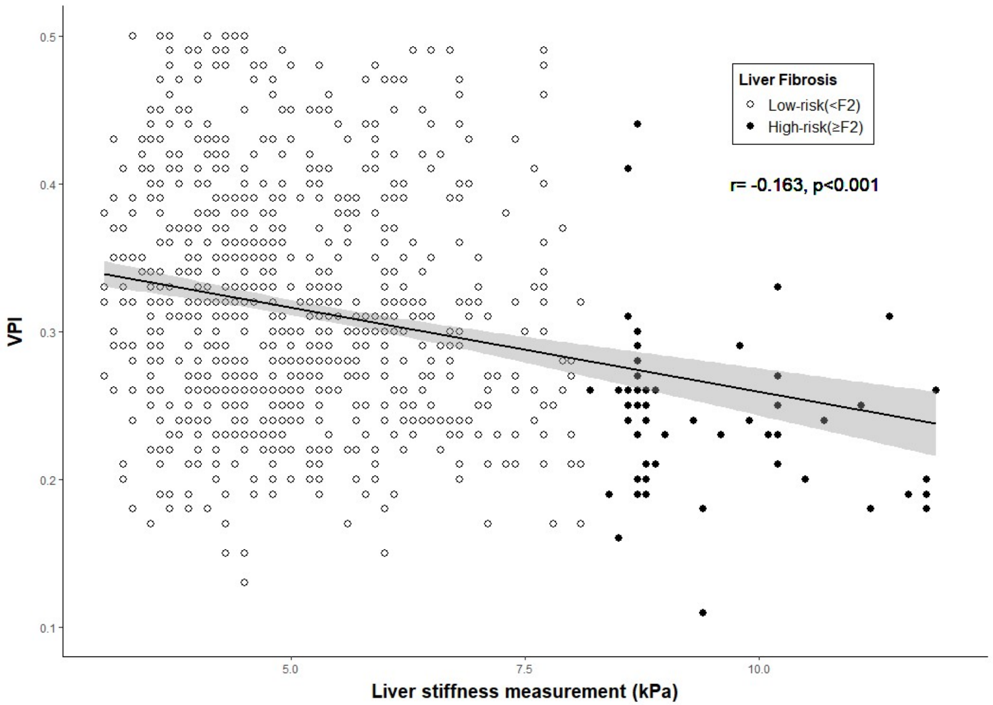
Liver Fibrosis
Low-risk(<F2)
High-risk(≥F2)
r= -0.163, p<0.001
VPI
0.5
0.4
0.3
0.2
0.1
5.0
7.5
10.0
Liver stiffness measurement (kPa)

(**A**)

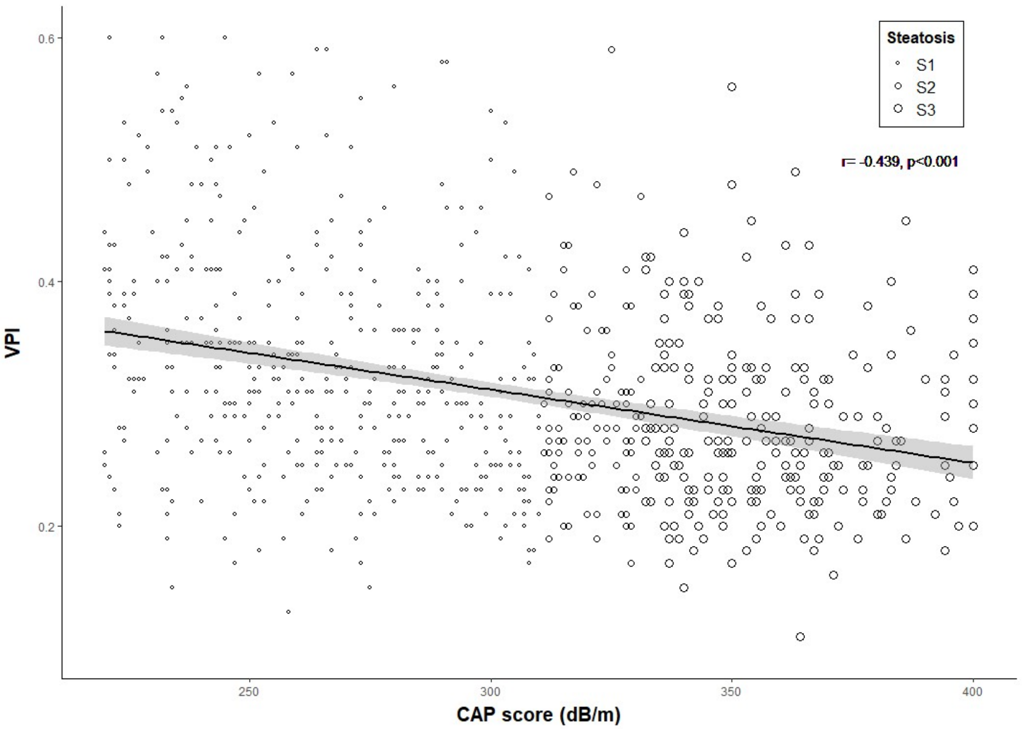

(**B**)

Figure S1. Scatter plot and linear regression line demonstrating correlation between VPI and CAP or LSM (**A**) VPI and LSM. (**B**) VPI and CAP. Abbreviations: CAP, controlled attenuation parameter; LSM, liver stiffness measurement; VPI, portal venous pulsatility index.

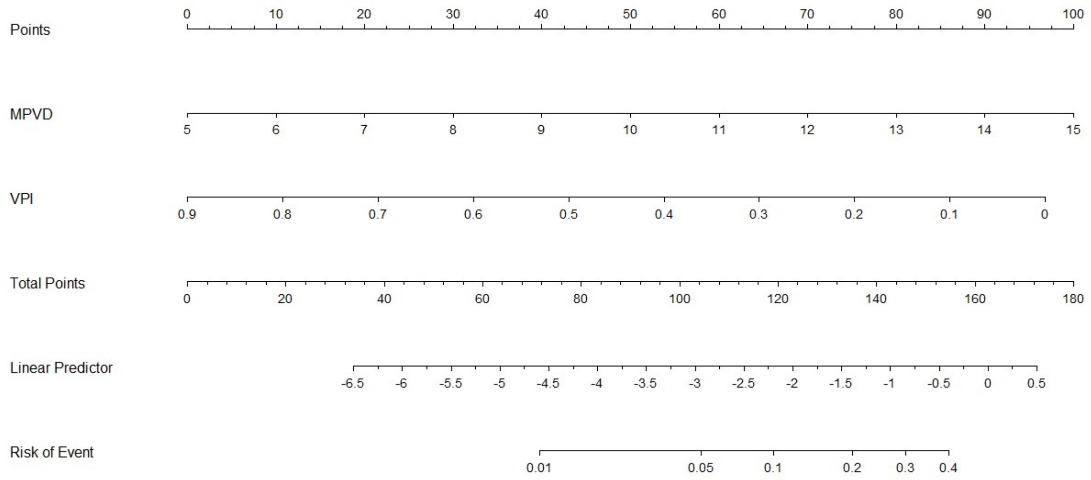

Figure S2. Nomogram for predicting significant fibrosis. Abbreviations: MPVD, main portal vein diameter; VPI, portal venous pulsatility index.
